# Optimization of entrapment efficiency and release of clindamycin in microsponge based gel

**DOI:** 10.1038/s41598-021-02826-7

**Published:** 2021-12-02

**Authors:** ALAA KHATTAB, Abdulhakim Nattouf

**Affiliations:** Faculty of Pharmacy, Damascus University, Damascus, Syria

**Keywords:** Diseases, Nanoscience and technology

## Abstract

The aim of the present study was to formulate clindamycin (CLN) as a microsponge based gel to release the drug in a controlled manner and reduce the side effects in the treatment of acne. Since this method requires poor water solubility of the drug to be loaded in particles, therefore, conversion of the hydrochloride salt to free base was done. By using an emulsion solvent diffusion method, we made six different formulations of microsponges containing CLN-free base by changing the proportions of polymer, emulsifier and the pH of the external phase. These formulations were studied for physical characterization and for drug- polymer interactions. The physical characterization showed that microsponge formulations coded by C5, C6 resulted in a better loading efficiency and production yield and their particle size was less than 30 µm. Scanning electron microscopy images showed the microsponges porous and spherical. C5, C6 microsponge formulation was prepared as gel in Carbopol and in vitro evaluated. The microsponge formulation gel C8 was found to be optimized. C8 released 90.38% of drug over 12 h and showed viscosity 20,157 ± 38 cp, pH of 6.3 ± 0.09 and drug content of 99.64 ± 0.04%. Fourier transform infrared spectroscopy and differential scanning calorimetry confirmed no significant interactions between excipients and drug.

## Introduction

Clindamycin (CLN) is a potent lincosamide antibiotic against susceptible anaerobic bacteria and gram-positive aerobes. It exhibits bacteriostatic action by binding to the 50 s subunit of bacterial ribosomes and inhibiting protein synthesis. At high concentrations, CLN may exhibit bactericidal activity^1^. Furthermore, CLN also has anti-inflammatory activities^2^. This medication is administered orally or parenterally to treat infections of the lungs, skin, blood, female reproductive organs, and internal organs. In addition, it is applied topically in the management of acne vulgaris^1^.

Acne vulgaris is a widespread follicular skin disorder that typically affects the Pilosebaceous unit of the face and upper part of the body and is triggered by different factors involving follicular hyperkeratosis, excess sebum production, Propionibacterium acnes hyperproliferation and inflammation. These factors produce non-inflammatory or inflammatory lesions that can be serious and leave scars^3^. Therefore, acne may have severe psychosocial consequences resulting in social isolation, emotional problems and depression. Topical treatment is the first line therapy for acne, and CLN, as a gel or a solution, is one of the most common and efficacious topical anti acne agents. Due to anti-bacterial and anti-inflammatory properties of CLN, it targets two key factors in acne pathogenesis: P. acnes colonization and inflammation^4^. However, conventional topical formulations of CLN immediately release the drug upon application in excessive concentrations over a short period of time, and then they release the drug in relatively low concentrations. Thus, resulting in two critical problems, the first one is cutaneous side effects such as irritation, dryness, oiliness, itching, erythema and peeling. These side effects may be serious and represent a troublesome issue for acne patients who have been afflicted psychologically and already suffering from inflamed lesions^5^. The other problem is the short duration of drug action, thereby, the drug should be applied two times daily^1^. In addition, CLN may have unpleasant odor which cannot be masked via commercial products^6^. As a result, limitations of conventional topical preparations of CLN may lead to poor patient compliance and reduction in treatment efficacy. Therefore, it is prudent to develop a new technique for delivering CLN that overcomes the disadvantages of its marketed products.

Microsponge technology ideally addresses the problems discussed above due to its several attractive features. Microsponges are polymeric, porous microspheres entrapping the active ingredients inside their tiny voids. These microparticles are retained in the crannies of the skin without passing through it and gradually release proper concentrations of the drug at the targeted site for a prolonged period. Thus, microsponges would control the drug delivery, enhance the drug tolerability, and reduce the frequency of application while still sustaining the efficacy of the drug. In addition, they could absorb sebaceous secretions which are highly found on the skin of most acne patients without cause drying. Furthermore, they may encapsulate the drug and mask its undesirable characteristics. As a result, microsponges would improve patients’ acceptance and adherence to the treatment, as well as maximize the therapeutic outcomes^7,8^. Based on the previous facts, we aimed in the present work to design a sustained release topical formulation of CLN by incorporating CLN into microsponges and dispersing CLN loaded microsponges into Carbopol gel as a vehicle. However, CLN is commercially available as CLN-HCl and CLN-PO4, Both of which are freely soluble in water, and it is a challenging task to incorporate them into microsponges via quasi emulsion solvent diffusion method, as high amounts of the freely soluble drug would leach into the external aqueous phase during formulation and lead to low drug entrapment^9^.

Thus, we attempted in this study to use CLN-free base (the slightly soluble form of the drug) instead of CLN-HCl or CLN-Po4 and change pH of the external aqueous phase as a dual approach in order to maintain the drug in the internal phase during the formulation and prevent its leaching into the external aqueous phase, which could, in turn, achieve maximum drug entrapment. To the best of our knowledge, this study presents the first attempt to use the dual approach mentioned above for formulating highly soluble drugs like CLN-HCl into microsponges, and the first attempt to develop sustained release microsponge based gel of CLN-free base.

## Materials and methods

### Materials

Clindamycin HCl was purchased from Sigma-Aldrich (Germany). Polyvinyl Alcohol was obtained from HiMedia labs (Mumbai). Carbopol 934 was bought from Loba chemie (India). Ethyl cellulose was obtained from Sigma-Aldrich (Germany). Dichloromethane, Ethanol, Sodium hydroxide and HPLC solvents were of analytical grade and purchased from Merck (Germany).

### Methods

#### CLN-HCl conversion from hydrochloride salt to base

CLN-HCl was converted to the corresponding free base by treatment with NaOH as previously described^10^. The detailed methods were as follows. A CLN-HCl solution was prepared by dissolving 57.1 g of CLN-HCl in 170 mL of deionized water in a 500 mL beaker. About 120 mL of 1.0 N NaOH was slowly added to the solution. The solution became cloudy and large white sticky ball-like lumps formed at the bottom of the beaker. The solution mixture in the beaker from the preceding step was stirred by glass rod until the ball-like lumps were manually deaggregated. After deaggregation, the ball-like lumps became small white solid species and were precipitated in the solution, which indicated CLN-free base was formed. Finally, the precipitate was dried by vacuum drier and stored before use.

#### Preparation of CLN-free base microsponges

The microsponges enclosing CLN-free base were fabricated by quasi-emulsion solvent diffusion method. The method consisted of preparing two phases: the inner phase and the outer phase. The inner phase comprises ethylcellulose (as mentioned in Table 1) dissolved in 20 mL of DCM. Once a clear solution was obtained, the CLN-free base was added and dissolved through ultrasonication at 25 °C for 30 min to obtain a homogenous clear solution. Then, this mixture was added dropwise into an aqueous solution of PVA (outer phase) with a stirring rate 1200 rpm for 3 h. Subsequently, microsponges were formed due to the DCM removal from the system by evaporation. The prepared microsponges were then filtered, washed with distilled water, and subjected to drying at 40 °C for 24 h in a hot air oven^11^. As shown in Table 1, various formulation batches were prepared.

**Table 1:** Formulae for microsponges of CLN-free base.

Ingredients	C1	C2	C3	C4	C5	C6
CLN free base (mg)	100	100	100	100	100	100
Ethyl cellulose (mg)	200	300	300	200	200	300
Dichloromethane (ml)	20	20	20	20	20	20
PVA (mg)	200	200	300	300	200	300
Distilled water (ml)	150	150	150	150	–	–
NaOH (0.01 N) (ml)	–	–	–	–	150	150

#### Evaluation of microsponge

1. Production yield and entrapment efficacy
  The entrapment and yield of the microsponge system was calculated using the following equations^12^:
  1 $${\text{Production yield }}\% = \frac{weight\;of\;the\;dried\;microsponges }{{sum\;of\; the\;initial\;dry\;weight\;of\;starting\;materials}} \times 100$$
  2 $${\text{Entrapment efficacy }}\% = \frac{actual\;drug\;content }{{theoretical\;drug\;content }} \times 100$$
2. Particle size evaluation
  The average particle size of CLN loaded microsponges was determined with an optical microscope using a calibrated ocular and stage micrometer.
3. Scanning electron microscope (SEM)
  The microsponge formulation C6 was visualized by SEM to assess the morphology and surface characteristics of the microsponges. (SEM, TESCAN, VEGA, Czech republic)^13^
4. Preparation of CLN-free base microsponge gel
  Gel of CLN-free base is prepared by using the following formula given in Table 2. A clear dispersion of Carbopol 934 was prepared in mixture of water and glycerine using moderate agitation. Parabens and Edetate disodium were dissolved in water and added to the previous mixture. Triethanolamine was used to neutralize the obtained viscous solution with slow agitation. At this stage, CLN-free base microsponges (equivalent to 1% w/w of CLN-free base) were incorporated to obtain homogenous CLN microsponge-loaded gels.

**Table 2:** Formulae for microsponge loaded gels of CLN-free base.

Ingredients	C7	C8
Microsponges (mg)	C5 formula equivalent to 1 g of drug	C6 formula equivalent to 1 g of drug
Carbopol 934 (g)	0.3	0.3
Glycerine (g)	5	5
Triethanolamine	q.s	q.s
Methyl paraben (g)	0.18	0.18
Propyl paraben (g)	0.02	0.02
EDTA (g)	0.05	0.05
Distilled water (g)	q.s.100	q.s.100

#### Gel evaluation

1. Appearance
  The formulated gels were examined visually for their color, appearance, and consistency.
2. Determination of pH
  The pH of the formulation was determined by using a digital pH meter (HANNA 211).
3. Determination of viscosity
  The viscosity of the formulation was studied by a digital viscometer (Rotary viscometer STS-2011) using spindle R7 at 25 ± 10 °C.
4. Determination of drug content
  One gram of formulation was diluted with 100 mL of ethanol 95% with proper mixing. The solution was filtered and was analysed for drug content by HPLC method with proper dilution of the sample.
5. In vitro drug release studies
  In vitro release studies were performed using cellulose nitrate membrane. For this experiment, a vertical Franz diffusion cell with a surface area of 2.54 cm^2^ and a reservoir capacity of 9.5 mL was used. The membrane was placed between the two halves of the diffusion cell. The receptor compartment contained a mixture of water and ethanol (50:50, v/v), and its temperature was maintained at 32 ± 0.1 °C and stirred continuously using a magnetic stirrer. Each formulation weighing 0.5 g of microsponge based gel was placed on the donor side. A total of 2 mL of the sample was withdrawn from the receptor compartment at definite time intervals and replaced with an equal volume of fresh receptor fluid. The aliquots were suitably diluted with the receptor medium and analysed by HPLC method^14–16^.
6. In vitro drug release kinetics
  To investigate the release mechanism of CLN-free base from the microsponge loaded gels, the release data was analysed using zero order, first order, Higuchi, Hixson-Crowell, and Korsmeyer-Peppas.
7. Stability study
  Optimized batches of CLN microsponge gels were monitored for up to 6 months at 40 ± 2°/75 ± 5% RH as per ICH guidelines^17^. At the interval of 1, 2, 3, 4, 5, and 6 months, samples were withdrawn and analysed to determine changes in appearance, pH, viscosity and drug content, and drug release^14^.

#### Compatibility study

1. Differential scanning colorimetry (DSC)
  The DSC for drug and formulation C6 was studied (DSC131, SETARAM, France). Accurately weighed samples were transferred to aluminium pans and sealed. Samples were run at a heating rate of 10 °C/min over a temperature range 25–450 °C in an atmosphere of nitrogen^18^.
2. Fourier transformer infrared spectroscopy (FTIR)
  To ascertain compatibility, FTIR spectra of CLN-free base and other excipients were recorded in KBr disc. (Bruker IR, Germany)^19^.

### Statistical analysis

The results represented are the mean of three determinations. Data was subjected to Student’s *t*-test. *P* < 0.05 was considered indicative of significance, using Microsoft Excel 2010.

## Results and discussion

### Evaluation of the formulated microsponge production yield% (PY%) and entrapment efficacy% (EE%)

**Figure 1:**
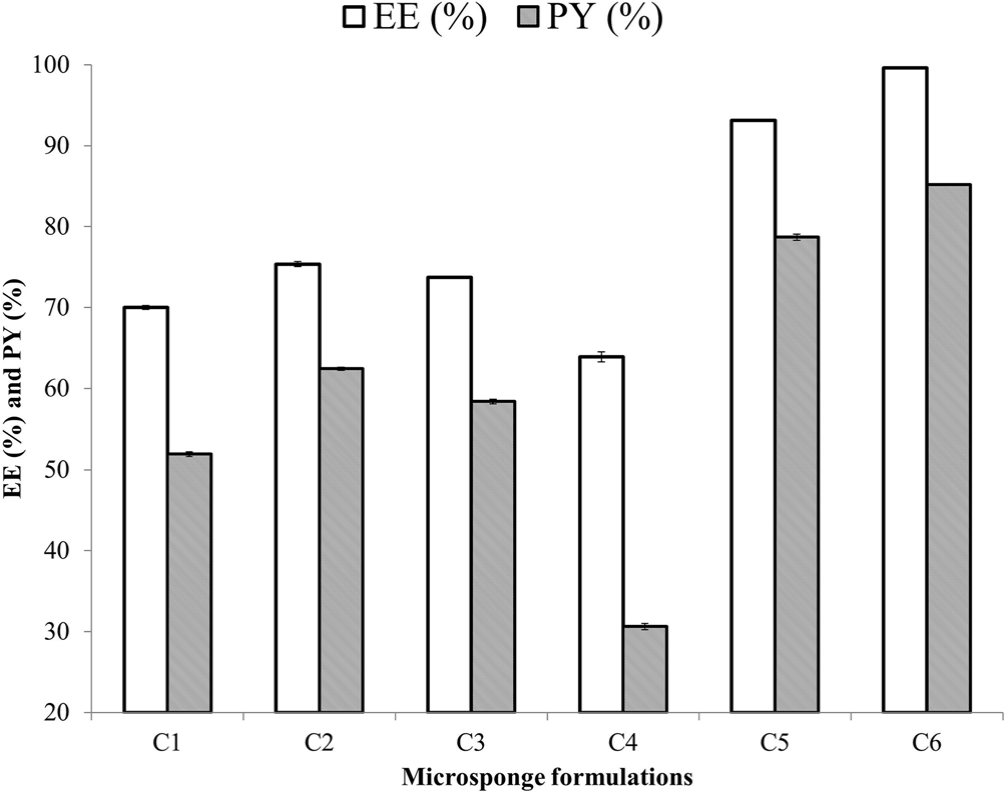
Graph Showing EE (%) and PY (%). Data are expressed as mean ± SD (n = 3).

The production yields and entrapment efficacy of CLN microsponge formulations are given in Table 3 and Fig. 1. All formulations revealed high EE% and PY%, which could be attributed to the porous structure of the microsponge formulations. Table 3 showed that the production yield and entrapment efficacy of microsponges significantly increased by increasing the amount of EC for a constant level of PVA, whereas increasing PVA concentration for a given value of EC produced microsponges with low EE% and PY%. These results can be explained by the high level of EC delays the diffusion of the organic phase to the aqueous phase due to higher viscosity of the organic phase. This provided more time for droplet formation and improved the yield and entrapment efficacy of the microsponges^20^. On the other hand, PVA is a nonionic emulsifier that forms hydrophobic regions, which dissolve some of the drug and polymer, thereby resulting in lower EE% and PY%^21^. To study the effect of pH on the entrapment efficacy and production yield of the prepared CLN-free base microsponges, distilled water at pH 5.8 and 0.01 N NaOH solution at pH 11 were used as an external phase. Microsponge formulations coded by C5, C6 were prepared as microsponge formulations coded by C1, C3 respectively, except that distilled water was replaced with 0.01 N NaOH solution. Results listed in Table 3 showed that increasing the pH of the external phase from 5.8 to 11 led to a significant increase in drug entrapment and yield. For example, The entrapment efficacy and production yield of CLN-free base were 73.72 ± 0.07% and 58.37 ± 0.27% respectively, with using distilled water at pH 5.8 as the outer phase, whereas they dramatically increased up to 99.61 ± 0.04% and 85.19 ± 0.01% respectively, by using 0.01 N NaOH solution at pH 11 as the external phase. This increased drug entrapment and yield is most likely due to a change in the degree of CLN-free base ionization. Namely, in 0.01 N NaOH solution at pH 11, CLN-free base was less ionized and therefore less soluble than in distilled water at pH 5.8. This may have therefore reduced partitioning of drug from the internal phase into the external aqueous and thus enhanced drug entrapment and yield into microsponges^22^.

**Table 3:** Results of CLN-free base microsponge EE% and PY%.

Formula	EE (%) ± SD	PY % ± SD
C1	70.02 ± 0.23	51.93 ± 0.29
C2	75.36 ± 0.31	62.42 ± 0.18
C3	73.72 ± 0.07	58.37 ± 0.27
C4	63.92 ± 0.62	30.64 ± 0.38
C5	93.15 ± 0.01	78.68 ± 0.39
C6	99.61 ± 0.04	85.19 ± 0.01

### Particle size

The particle sizes of CLN microsponge formulations are tabulated in Table 4. The particle size of CLN microsponges increased upon the increase in EC concentration. This could be explained by the fact that, at higher drug-polymer ratio, the available polymer was higher, thus increasing the polymer wall thickness, which led to increased size of the microsponges^23^. On the other hand, the particle size of CLN microsponges decreased upon the increase in PVA concentration. This was most probably due to the decrease in surface tension of the continuous phase by increasing the surfactant concentration, which results in smaller emulsion droplets and consequently smaller particle size^24^.

**Table 4:** Results of CLN-free base microsponge particle size.

Formula	Particle size (µm) ± SD
C1	21.92 ± 0.12
C2	33.73 ± 0.91
C3	25.17 ± 1.58
C4	15.26 ± 0.07
C5	23.47 ± 1.22
C6	26.49 ± 1.03

### Selection of optimized formulations

Microsponge formulations with particle size less than 30 µm (to avoid any gritty feeling) and maximum EE% and PY% (C5, C6) were considered as optimized formulations. Therefore, they were incorporated into gel base and further evaluated.

### Scanning electron microscope (SEM)

**Figure 2:**
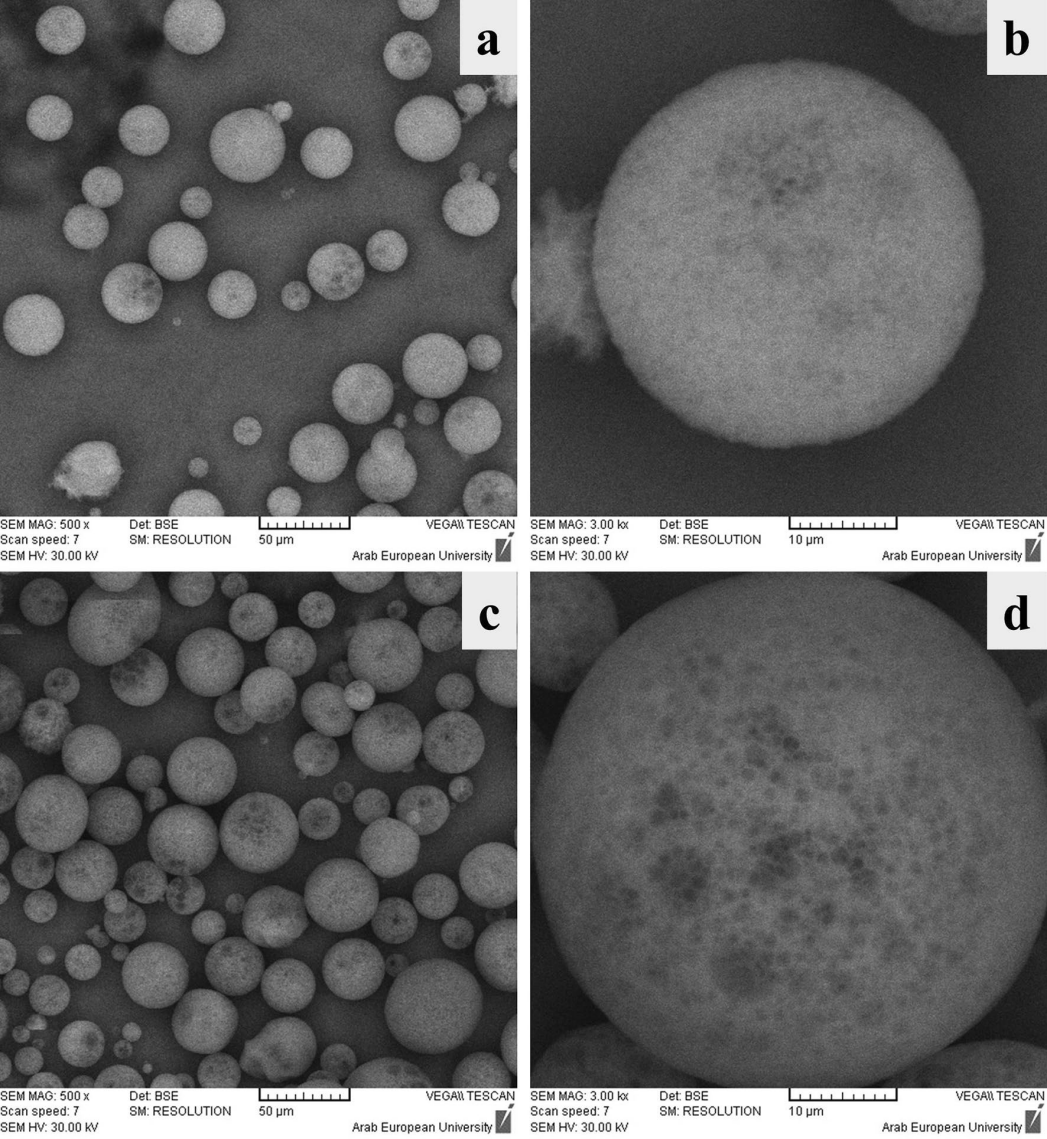
SEM images of (**a**) C5 formulation, 500x, (**b**) C5 formulation, 3000x, (**c**) C6 formulation, 500x, (**d**) C6 formulation, 3000x.

The representative SEM photographs of the microsponge formulations (C5, C6) are shown in Fig. 2. SEM images showed the microsponges were spherical and devoid of aggregation. Therefore, they would easily disperse into the gel formulation. In addition, the drug crystals were not seen on the surface of the microsponges. This indicates the dispersion of the drug inside the polymeric matrix which is interpreted in DSC analysis.

### Appearance and physical parameters of gels

The formulated gels were clear and transparent and showed the viscosity of 20,157 ± 38–21,911 ± 29 cps, pH of 6.3 ± 0.09–6.5 ± 0.1, and drug content of 99.64 ± 0.04 to 100.13 ± 0.29% respectively. pH values of all formulations are suitable for application to skin. The drug content of the formulations showed that the drug was uniformly distributed in the formulations. This is given in Table 5.

**Table 5:** Results of appearance and physical parameters of CLN-free base gels.

Formula	pH ± SD	Viscosity (cps) at 25 °C	Drug contnt (%) ± SD
C7	6.5 ± 0.1	21,911 ± 29	100.13 ± 0.29
C8	6.3 ± 0.09	20,157 ± 38	99.64 ± 0.04

### In vitro drug release studies

**Figure 3:**
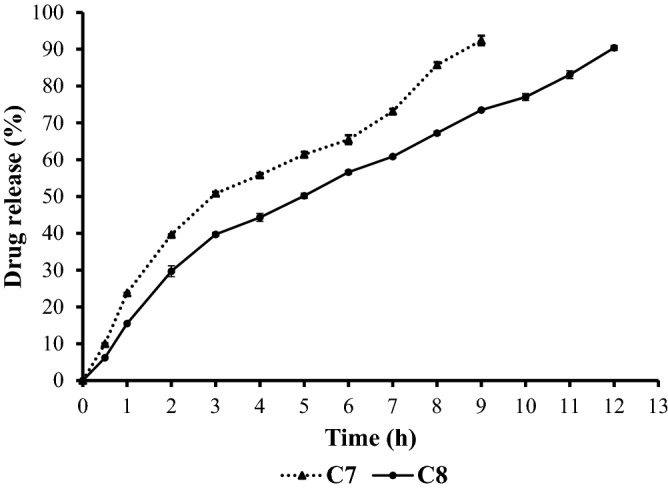
Percentage drug release of CLN-free base from C7 and C8 formulations against time (h). Data are expressed as mean ± SD (n = 3).

The in vitro performance of CLN-free base microsponge gels showed prolonged release of CLN. The results of the in vitro drug release studies of formulations C7 and C8 are shown in Fig. 3. The results of C8 formulation prepared with 300 mg EC showed a release of 90.38% in 12 h, while C7 formulation prepared with 200 mg EC showed a release of 92.39% in 9 h. Thus, the extent of drug release depended on EC levels. This could be explained by the significant increase in particle size of microsponges by increasing polymer concentration, which leads to a longer diffusion path, and consequently a slower release rate^25^. In addition, no initial burst release was seen during the first hours of drug release from both gel formulations. The reason behind that could be the absence of non-encapsulated CLN on the surface of the microsponges^19^. This finding is in agreement with SEM and DSC studies results. Data obtained from in vitro release studies were utilized for release kinetics, and the results of the in vitro drug release kinetics were shown in Table 6. The in vitro drug release of the formulations C7 and C8 were best explained by Higuchi kinetic model as the plots showed linearity R^2^ = 0.983 and R^2^ = 0.991, respectively, which are shown in Table 6. In the two formulations, n was found to be more than 0.5, indicating the diffusion was Anomalous diffusion (Non-fickian diffusion)^26^.

**Table 6:** Results of the in vitro drug release kinetics of C7 and C8 formulations.

Formula	Zero R^2^	First R^2^	Highochi R^2^	Peppas		Hicxon R^2^
				R^2^	n	
C7	0.955	0.906	0.983	0.965	0.7	0.846
C8	0.964	0.942	0.991	0.955	0.7	0.831

### Stability study

The results of the stability study of formulation C8 displayed no considerable changes in the physical appearance, pH, and drug content. Moreover, there were no significant changes in percent drug release. After comparing the drug release profiles of formulation C8 before and after 6 months stability study, the similarity factor (f2) was calculated, which was found to be f2 = 83.29 (> 50), which indicates good stability of the formulation. Therefore, the formulation C8 was established as stable over the period of 6 months^27^. Results were shown in Table 7.

**Table 7:** Results of stability study of C8 formulation.

Time (months)	Physical appearance	pH ± SD	Drug content (%) ± SD	CDR (%) ± SD
0	–	6.3 ± 0.09	99.64 ± 0.04	90.38 ± 0.02
1	No change	6.4 ± 0.01	98.28 ± 0.03	89.36 ± 0.04
2	No change	6.3 ± 0.04	99.45 ± 0.02	88.25 ± 0.03
3	No change	6.3 ± 0.02	99.26 ± 0.02	88.18 ± 0.08
4	No change	6.5 ± 0.07	98.48 ± 0.03	89.7 ± 0.04
5	No change	6.5 ± 0.02	97.84 ± 0.03	89.39 ± 0.07
6	No change	6.4 ± 0.03	97.23 ± 0.03	90.32 ± 0.03

### Compatibility study

#### Differential scanning colorimetry (DSC)

**Figure 4:**
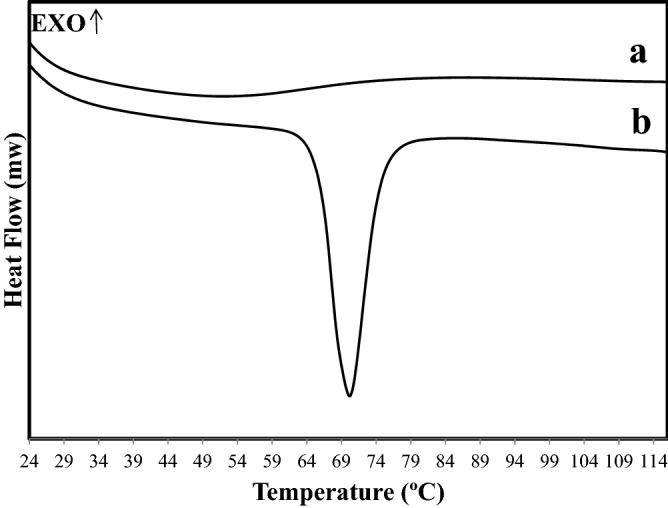
DSC thermograms of (**a**) pure CLN-free base, (**b**) C6 formulation.

To investigate the thermal behaviour of CLN-free base in the formulated microsponge, DSC study was conducted and the thermograms of CLN-free base, and microsponge formulation C6 are illustrated in Fig. 4a,b respectively. DSC thermogram of pure drug showed a sharp endothermic peak at 70.21 °C, indicating that drug crystals had melted^10^. On the other hand, the thermogram of CLN-free base microsponge formulation C6 in Fig. 4b exhibited a detectable loss of the melting endothermic peak of pure CLN-free base. This clearly indicated that the drug was dispersed homogeneously throughout the microsponges and these microsponges had an amorphous structure as all cross-linked polymers^28,29^.

#### Fourier transform infrared spectroscopy FTIR

**Figure 5:**
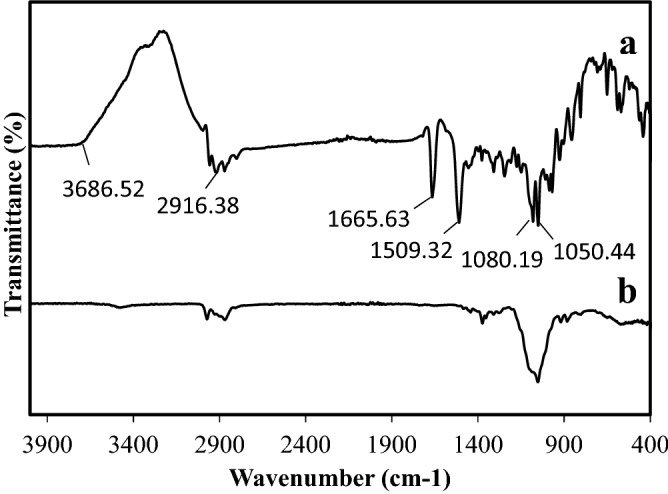
FTIR spectra of (**a**) pure CLN-free base, (**b**) C6 formulation.

The IR spectra of CLN-free base pure powder and microsponge formulation C6 are as shown in Fig. 5a,b, respectively. CLN-free base spectrum shows peaks at the following locations: at 3686.52 cm^−1^ due to O–H stretching in the galactose sugar group; at 2916.38 cm^−1^ due to C–H stretching of C4-alkyl group; at 1665.63 cm^−1^ and 1509.32 cm^−1^ related to N–C=O stretching of amid carbonyl group; at 1080.19 cm^−1^ corresponds to C–O cyclic ether stretching in the galactose sugar group; at 1050.44 cm^−1^ due to C–C stretching of pyrrolidine group^30,31^. The IR spectrum of microsponge formulation C6 is totally different from CLN-free base powder. The characteristic bands of CLN-free base either have disappeared or reduced in intensity, which might be due to the restriction inside the microsponge matrix. In the infrared spectra of CLN-free base microsponge formulation C6, no additional peak was observed, which emphasized the absence of any possible interaction between CLN-free base and other excipients^32^.

## Conclusions

The aim of this study was to develop a sustained release microsponge based gel of CLN-HCl as a model for a highly water-soluble drug with high entrapment efficiency of the drug inside microsponges and acceptable other physicochemical properties. Initially, CLN-HCl was converted to CLN-free base. Then four formulations of CLN-free base loaded microsponges, C1, C2, C3 and C4 were prepared by quasi emulsion solvent diffusion method using two levels of EC (200 mg, 300 mg) and two levels of PVA (200 mg, 300 mg) and evaluated for their yield, entrapment efficacy and size. In an attempt to obtain microsponge formulations with higher yield and entrapment efficacy, C5 and C6 formulations were prepared by changing pH of the external aqueous phase from 5.8 to 11 via using 0.01 N NaOH solution instead of distilled water. C5 and C6 formulations had significantly high yield and entrapment efficacy as well as they had a particle size less than 30 µm suitable for dermal application. In addition, SEM images of these formulations revealed that microsponges were spherical, devoid of aggregation, and had a smooth surface without any free drug content. Further, C7 and C8 formulations were prepared by incorporating microsponge formulation C5 and C6 into Carbopol gel respectively and evaluated for appearance, viscosity, pH, drug content, In vitro drug release, In vitro drug release kinetics. Each of C7 and C8 formulations was clear and transparent and had acceptable viscosity, pH and drug content. In vitro drug release study showed that both of C7 and C8 formulations extended the drug release for a longer time without any burst release effect. However, C8 formulation exhibited a little superiority over C7 formulation since it released the drug up to 12 h. After the stability testing for a period of six months, C8 formulation was established as stable. In addition, FTIR and DSC studies indicated drug-polymer compatibility and amorphous nature of the microsponges structure. Eventually, microsponge gel formulations of CLN-free base were successfully developed with notable high entrapment efficiency and precise controlled release. Therefore, these formulations can be considered as promising delivery systems for CLN, by reducing side effects, decreasing frequency of application, enhancing aesthetic properties and improving patient compliance. On the other hand, this study opens the door for further consideration of the potential incorporation of CLN-free base loaded microsponges into other vehicles such as tablets or powders for systemically administration in order to get several benefits (e.g. masking the extremely bitter taste of CLN or decreasing its frequent administration). In addition, the above approaches, of converting CLN-HCl to CLN-free base and altering the aqueous phase pH, provide future insights for developing sustained release microsponges of similar water soluble-drugs.

## Data Availability

Correspondence and requests for materials should be addressed to A.K.
